# Efficacy of a Simplified Feedback Trainer for High-Quality Chest Compression Training: A Randomized Controlled Simulation Study

**DOI:** 10.3389/fpubh.2021.675487

**Published:** 2021-10-14

**Authors:** Xiao-yan Meng, Jia You, Li-li Dai, Xiao-dong Yin, Jian-an Xu, Jia-feng Wang

**Affiliations:** ^1^Department of Anesthesiology, Eastern Hepatobiliary Surgical Hospital, Naval Medical University, Shanghai, China; ^2^Department of Anesthesiology, Changhai Hospital, Naval Medical University, Shanghai, China; ^3^Department of Colorectal Surgery, Changhai Hospital, Naval Medical University, Shanghai, China

**Keywords:** cardiac arrest, chest compression, real-time feedback, external cardiac massage, quality management in cardiopulmonary resuscitation

## Abstract

**Background:** The most recent international guidelines recommended support training of chest compression (CC) using feedback devices. This study aimed to compare the training efficacy of a simplified feedback trainer with the traditional cardiopulmonary resuscitation (CPR) simulator in CPR training.

**Methods:** A total of 60 soldiers were randomly allocated into three groups equally, trained with a simplified external cardiac massage (ECM) trainer named Soul Sheath^TM^ (SS) (SS group), a Resusci Anne manikin (RA group), or traditional simulation training with instructor feedback, respectively. After 7 days of training, the CPR skills were tested blindly in a 2-min assessment session. The primary outcome was the proportion of effective CC, and the secondary outcome included CC rate, depth, compression position, and extent of the release.

**Results:** The percentage of effective CC achieved in the SS group was comparable with the RA group (77.0 ± 15.52 vs. 77.5 ± 10.73%, *p* = 0.922), and significantly higher than that in the control group (77.0 ± 15.52 vs. 66.8 ± 16.87%, *p* = 0.037). Both the SS and RA groups showed better CC performance than the control group in terms of CC rate (SS group vs. control group, *P* = 0.032 and RA group vs. control group, *P* = 0.026), the proportion of shallow CC (SS group vs. control group, *P* = 0.011 and RA group vs. control group, *P* = 0.017). No difference between the SS group and RA group was found in all the CC parameters.

**Conclusions:** The simplified ECM trainer (SS) provides a similar efficacy to the traditional manikin simulator with feedback in CC training to improve the quality of CPR skills.

## Introduction

The quality of chest compression (CC) is the primary factor for “high-quality” cardiopulmonary resuscitation (CPR) after cardiac arrest (1). In the 2020 American Heart Association (AHA) guideline, rescuers are recommended to perform compressions to a depth of over 5 cm and a rate of 100–120 counts/min, and real-time feedback devices for quantifying CC quality are also highly recommended (2). Numerous clinical evidence has already proved that shallow CC, incomplete release, and inappropriate CC rate might result in poor coronary perfusion and low cardiac output. These inappropriate manipulations might be associated with a decrease in the survival rate of 30% or more after cardiac arrest (3–6). Moreover, although the over-deep compression was not emphasized in the new guidelines of AHA CPR, a compression depth deeper than 6 cm may develop other complications, such as fracture of the ribs, pneumothorax, and hemothorax (7–9). Thus, it is challenging but essential for rescuers to reach an ideal range of CC during CPR.

One approach for improving CC quality is to implement auditory-visual feedback devices with a built-in accelerometer to provide data regarding the CC depth, rate, and intensity (10, 11). Recently, with the improvement of devices, feedback systems are now including in many smartphones and smartwatches. According to a review by An et al. (12), a feedback system of smart devices could improve the parameters of CC performed by rescuers who have received CPR training.

Although the CPR skills can be improved by regular training with the help of the feedback systems and new devices, the CPR skills tend to be elapsed after a certain period after training, and the CPR performers need frequent practice to adhere to the recommended guidelines (13). Thus, a simplified external cardiac massage (ECM) training device named Soul Sheath^TM^ (SS) with real-time feedback (HeartFellow, Shanghai, China) was developed for daily training at home. In the present study, the training efficacy of the simplified trainer was compared with a traditional manikin simulator with feedback in a randomized controlled trial.

## Materials and Methods

### Study Design and Participants

We designed a prospective, randomized controlled study to evaluate the efficacy of the SS trainer in improving the CC quality. The participants were recruited from the volunteer soldiers. Exclusion criteria for this study were as follows: (1) wrist, spine injury, or pulmonary/heart diseases, or other medical contraindication to physical exercise; (2) with a previous professional CPR training or actual CPR experience; (3) refusal to the training or assessment or cannot attend for other reasons. The local Ethics Committee on Human Research (Changhai Hospital, second military medical university, Shanghai, China) stated that this was an educational trial, as approval was not necessary. All the participants received written information about the protocol and gave written consent for data acquisition and analysis.

The instructors were anesthesiologists, intensivists, or emergency physicians from Changhai hospital, all of whom were experienced in practicing CPR and had participated in CPR training courses in the preceding years. Before the start of the training period, all the instructors were also invited to participate in a meeting for updated information on emergency procedures and the training process, to avoid any confusion in the training details.

### Equipment and Materials

The present study used the Resusci Anne (RA) manikin (Skill reporter™; Laerdal, Stavanger, Norway) as a standard training device for basic training and assessment. The manikin, *via* a laptop program for simulation, could estimate and record various CC parameters, such as the proportion of positive compression, depth (recorded as the proportion of over/shallow compression in the 2-min assessment), rate, number of inappropriate hand position, and proportion of incomplete releases, with real-time visual and auditory feedback provided.

**Figure 1:**
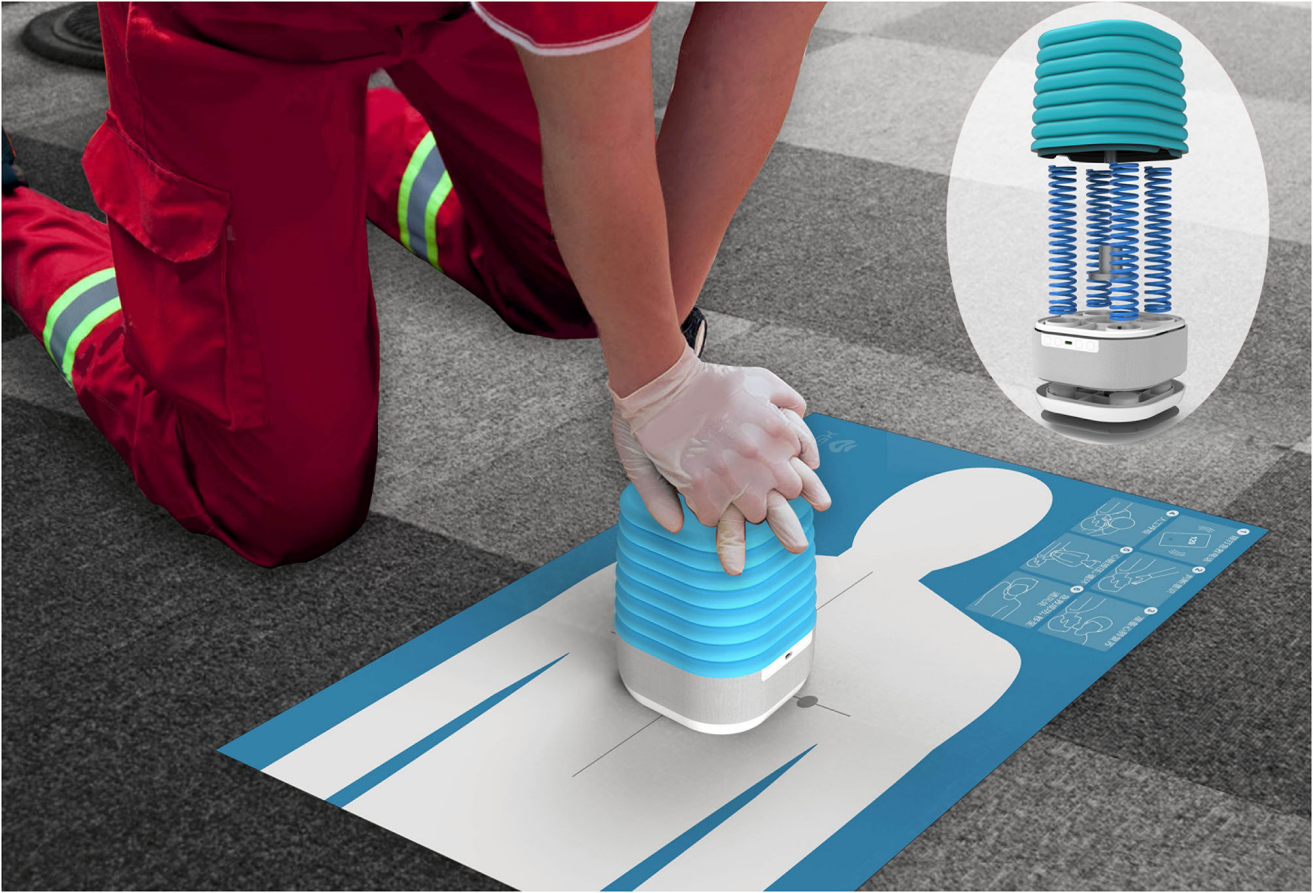
The schematic diagram for the Soul Sheath^TM^ training device. The device is composed of a cover made of silica gel, four springs and a base with a Bluetooth connector, and a sensor monitoring the distance of movement induced by the compression and the rate of compression. The speaker and the indicator light are also integrated into the base. There is also a sheet with a brief instruction of the cardiopulmonary resuscitation process and an upper half of a human shape to mark the position of chest compression.

The simplified SS ECM device is a Bluetooth speaker with a size of 40 cm × 20 cm × 20 cm and a built-in sensor to measure all the CC parameters mentioned above and provide auditory feedback. By connecting to a smartphone, real-time visual feedback could be provided and parameters could be recorded, such as practice time, rate, compression depth, and a voice warning of inappropriate release (Figure 1). The resistance value of the four springs in the device was determined by measuring the effort required to compress the commercially available RA manikin for 6 cm, which was about 35–45 kg. Thus, the resistance value of the four springs in the SS device was determined to be 40 kg. So that the strength needed to compress the SS device was similar to that needed for the RA device.

### Intervention

To minimize the heterogeneity in the study population, all the participants accepted 1 h of theoretical education and followed by a half-day CPR performance training using the RA manikins. Under the guidance of instructors, all the participants performed the procedural task several times for a total of 5–8 min, to ensure that they all mastered the basic CC skills.

After the training, the participants were randomized into three groups with a computer-generated list of random numbers (Microsoft Excel, Redmond, WA, USA). In the next 7 days, they were further trained separately in afternoon time according to the grouping. During the 7-day course, all the three groups were trained at the same time but in a separate classroom with two trainers for each group every other day. Each training lasted for 2–3 h to ensure that all the participants have experienced the CC procedure, and all the groups accepted the same total training time. The training was mainly focused on CC without any specific scenario simulation for cardiac arrest. All three groups are under the guidance and supervision of trainers, and they were also allowed to communicate and discuss freely.

In the control group (*n* = 20), the participants accepted the traditional CRP training process, by which the participants trained with CPR in a traditional CPR manikin with feedback from instructors. In the RA group (*n* = 20), the participants used the Resusci Anne system with real-time feedback of the CC parameters. After explaining to the instructor how to use the system and read the feedback CC parameters, the participants were trained and guided by the auditory and visual feedback under the supervision of the trainers. In the SS group (*n* = 20), the participants were trained with the SS device similarly to the RA group.

The participants of the three groups were blinded in the training methods and were trained separately. After 7 days of training, the CC performance was assessed. Each participant was tested in an independent classroom to perform a 2-min CC using the Resusci Anne device without real-time feedback. The final assessment was held by the two instructors who were blinded to the group allocation. The CC parameters and the demographic characteristics of the participants (age, weight, CPR education frequency, and experience of a real-life CPR situation) were recorded immediately after the assessment.

### Primary and Secondary Outcomes

The primary outcome was the proportion of effective CC within 2 min, which is defined as a successful CC with a depth that met an appropriate depth between 5 and 6 cm in a precise direction. Secondary outcomes, such as CC rate, the proportion of shallow CC, over CC, incorrect hand position, and ratio of complete release. All these parameters were recorded by the Skill reporter system and downloaded by an author who is blinded to the grouping.

### Statistical Analyses

All the statistical analyses were performed with SPSS 25.0 (IBM SPSS Statistics, Armonk, NY, USA). The normality of all the datasets was tested using Kolmogorov–Smirnov tests. The continuous variables were presented as the mean ± SD, while the categorical variables were presented as numbers and proportion (%). For continuous variables, one-way ANOVA with a *post-hoc* analysis of least significant difference (LSD) test or Tamhane's T2 test was used for data analysis, and the chi-square test was used for the categorical variables. The value of *P* < 0.05 was considered statistically significant.

For sample size calculation, with an α = 0.05, β = 0.2, we assumed a non-inferior margin of 2.5% for the rate of successful CC and an SD = 3% according to the reference (11), and the sample size should be 19 for each group by calculation *via* PASS 11 (UT, USA). Therefore, we recruited 20 subjects in each group.

## Results

**Figure 2:**
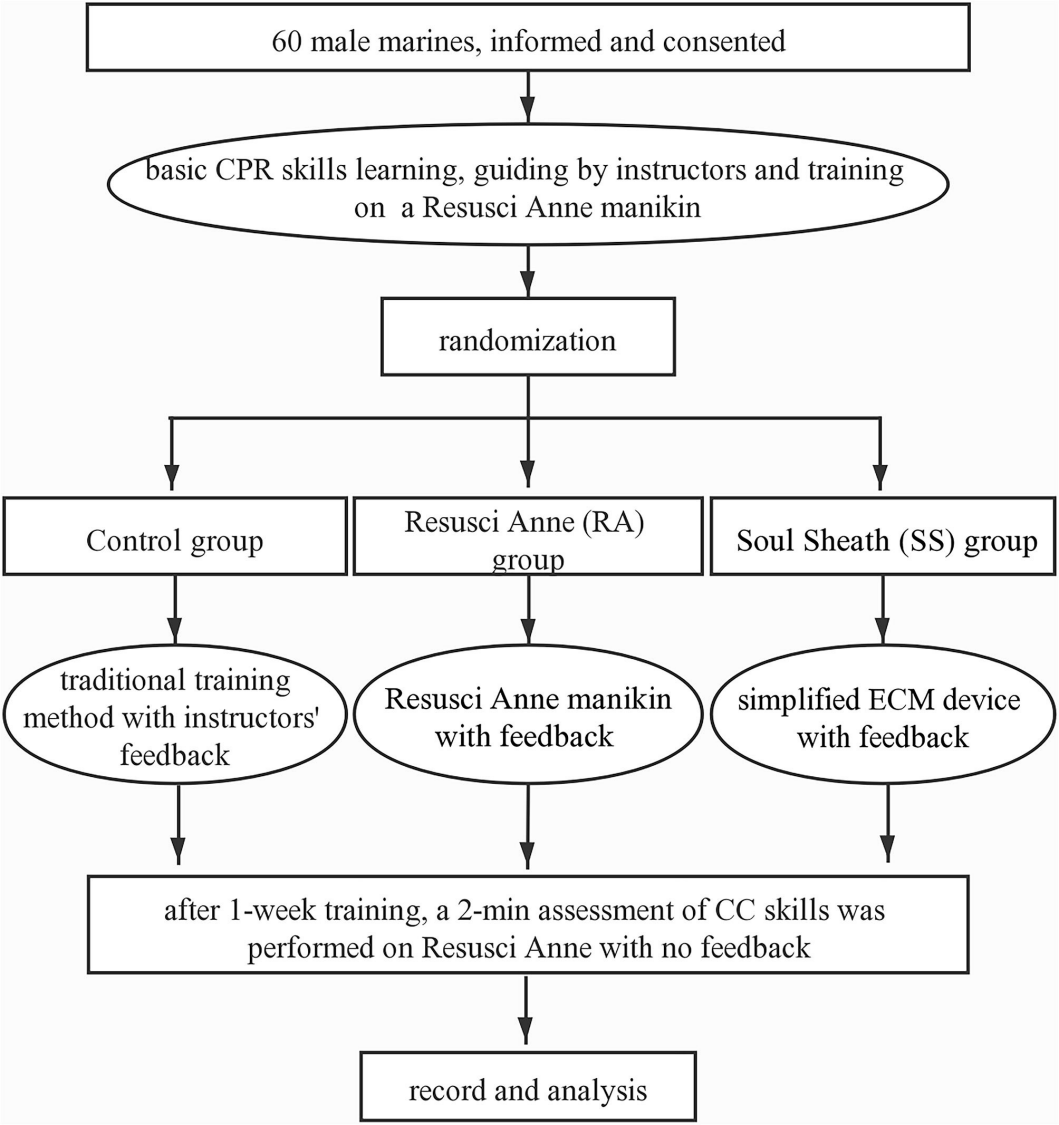
Flowchart. CPR, cardiopulmonary resuscitation; ECM, external cardiac massage; CC, chest compression.

**Figure 3:**
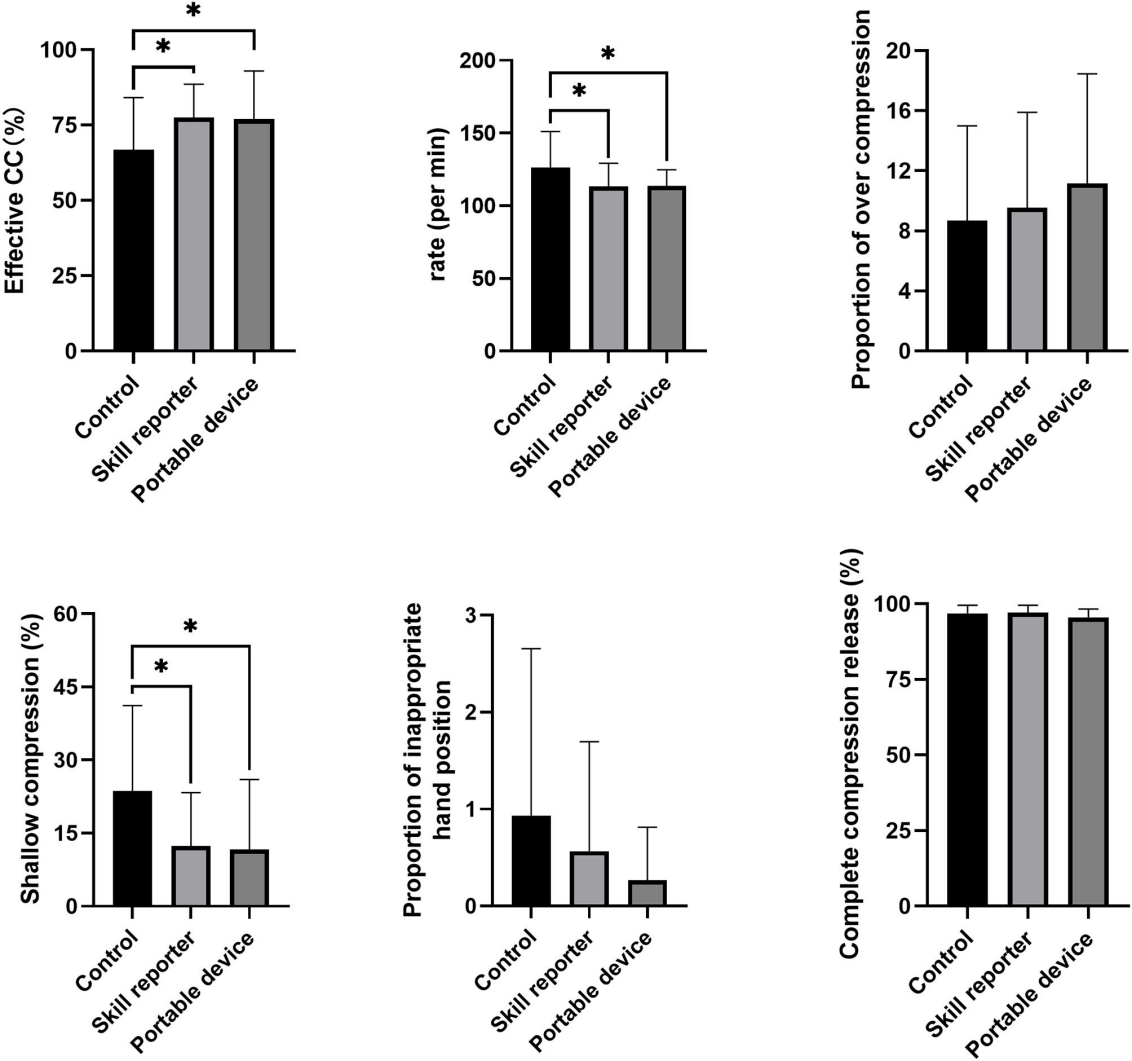
Chest compression scores in the three groups. A significant difference was present in the proportion of effective CC rate, CC rate, and proportion of shallow CC. The statistical comparisons are discussed in the text. ^*^p < 0.05.

All the 60 male soldiers fulfilled the inclusion criteria and were enrolled in the study. The study flow is presented in Figure 2. The baseline characteristics of the participants are summarized in Table 1. The average age for all the participants was 23.07 ± 2.65 years old and the average attending time was 4.96 ± 2.18 years. No significant difference was present in the demographic data among the three groups. The proportion of effective CC of the control group was 66.8 ± 16.87%, while the proportions were much higher in the RA group (77.5 ± 10.73%, *p* = 0.028 vs. control group) and SS group (77.0 ± 15.52%, *p* = 0.037 vs. control group and *p* = 0.906 vs. RA group) (Figure 3). In terms of CC rate, the participants in the RA group and SS group showed similar results (113.2 ± 15.69 vs. 113.6 ± 10.90 counts/min, *p* = 0.940), while both the two feedback groups showed superiority over the control group (126.3 ± 24.20 count/min, *P* = 0.026 vs. RA group and 0.032 vs. SS group, respectively). Significant difference was also present in the proportion of shallow CC, when comparing control (23.6 ± 10.09%) with SS group (11.6 ± 9.67%, *p* = 0.011) and with RA group (12.4 ± 10.68%, *p* = 0.017 vs. control group and *p* = 0.868 vs. SS group). For more secondary outcomes, no significant difference was found among the three groups (Figure 3).

**Table 1:** Demographic characteristics.

**Variable** **(mean ± std)**	**Control group** **(*n* = 20)**	**RA group** **(*n* = 20)**	**SS group** **(*n* = 20)**	***p*-value**
Age (years)	23.0 ± 2.35	23.0 ± 2.35	23.1 ± 3.53	0.554
Attended time	5.0 ± 2.10	5.0 ± 2.09	4.8 ± 2.83	0.759
Height (cm)	176.9 ± 7.15	173.1 ± 4.27	173.2 ± 5.79	0.115
Weight (kg)	71.7 ± 9.09	70.2 ± 11.23	70.1 ± 6.54	0.132
BMI	23.3 ± 3.18	23.3 ± 3.05	23.3 ± 1.83	0.229
Education background				0.158^*^
High school	7 (35%)	4 (20%)	2 (10%)	
Junior college	13 (65%)	15 (75%)	15 (75%)	
Bachelor	0	1 (5%)	3 (15%)	

*RA, Resusci Anne manikin; SS, Soul Sheath device; BMI, body mass index; std, standard deviation*.

* *P = 0.156 for SS vs. Control group; P = 0.432 for SS vs. RA group; and P = 0.375 for RA vs. Control group, chi-square test followed by Bonferroni correction*.

## Discussion

The main finding of this study is that the simplified SS trainer shows a comparable training efficacy in improving the CC quality with the traditional RA manikin device with a feedback function. The training efficacy of SS and RA devices with feedback function is much superior to training manikin without feedback function in terms of the ratio of successful CC, CC rate, and the ratio of shallow CC.

The quality of CC remains the requisite for improving the survival rates of cardiac arrest patients (1, 5). The effective CC components, such as hand position, the position of rescuer and victim, compression rate, and depth. Among these components, the correct CC depth and rate according to the guidelines of AHA are more difficult to maintain, as the previous studies have demonstrated that a relatively large percentage of medical care personnel in the United States have difficulty in performing them properly. According to the recent international guidelines, a compression rate of 100–120 count/min and a depth >5 cm are beneficial for improving the patient outcomes, while compressions at a rate of >120 counts/min or at a depth of <38 mm may result in poor coronary perfusion and low cardiac output and eventually lead to poor prognosis. Noting that in the most recent guideline of AHA, a CC depth of >5 cm has been recommended, rather than at a range of 5–6 cm (2, 3). This change in the guideline implies a growing concern on the harm of shallow CC, rather than the complications induced by over CC.

To improve the consistency and quality of the CC depth and/or rate of rescuers, many devices with real-time feedback systems have been developed and tested in previous years (14, 15). Recently, a Spanish study reported that the laypeople who received brief training with real-time feedback devices could provide similar CC performance to the health professionals (16). Another study proved that two feedback training devices (one of them was Resusci Anne equipped with Skill reporter software) improved the quality of external cardiac massage skills when compared with the traditional teaching in medical students (17), and more evidence corroborated this conclusion in other populations, such as laypeople, nurses, and professional persons (18–20). Our results are in line with these results that the feedback device–only training is feasible and is associated with improved ECM quality. Moreover, with the wide use of smart devices, a feedback system based on the use of smart devices to improve the quality of CC has emerged and tested in manikin studies (12). Our results add more evidence on the benefits of involving smartphones in CPR monitoring. Indeed, when comparing the training efficacy of the two feedback devices, we showed that they are both associated with more stable CC frequencies and less amount of shallow CC when comparing with the traditional training, while no difference in training performance was found between the simplified SS device and RA manikin device with feedback. Herein, our results showed that both the two feedback devices were suitable for CPR training in place of traditional instructor-guided training method. Noting that the benefits of real-time feedback on CC quality was assessed based on mainly manikin studies, and therefore whether the real-time CPR feedback devices could improve the patient outcomes in real-life resuscitations remain to be determined.

The previous reports implied that even for the well-trained rescuers, the decline in ECM skills remained to be inevitable several months after training (21, 22). In this regard, this kind of simplified training device might be more superior to the traditional manikins because the portable design may facilitate the laypeople to maintain a longstanding self-training in a nonprofessional environment such as a home. The cost of the SS device has not been determined yet, but the price would be much cheaper than commercially available simulating manikin with a feedback function according to the manufacturer. Therefore, this SS device is simple, portable, and economic equipment, which can provide a similar training effect in CC compared with the classical RA training machine. As an alternative training device for CPR, it has prominent advantages at repetitive self-training of ECM skills, ease of use in skilled rescuers or laypeople, and convenience for at-home training or outdoor training. However, it is not designed for training in other domains of the CPR skills, as other auxiliary equipment for better simulation of real-world resuscitation situations is needed.

Several limitations of this study should be addressed. First, all the participants were male soldiers with young age and a relatively small sample size. Although this population provides convenience in randomization and blinding owning to discipline among the servicemen, this may limit the application and explanation of the results. Second, this study was a simulation study using manikins, whether the improvements of training results on these manikins are associated with clinical benefits remain undefined. Third, the SS device with an accelerometer is designed for ECM training only, while the CPR needs more skills in judgment and breathing, which cannot be trained based on the current device. Moreover, the current study involves a 1-week training, followed by an immediate assessment. The effect of the feedback devices on long-term outcomes or the benefit in maintaining persistent CPR skills remains uninvestigated. In this regard, further clinical studies on the cardiac arrest patient outcomes, such as the return of spontaneous circulation, are warranted.

## Conclusion

The use of the simplified SS device provides a non-inferior training efficacy to a traditional manikin device with feedback in improving the performance of CPR in terms of successful compression ratio, correct compression rate, and the prevention of shallow compression.

## Data Availability Statement

The original contributions presented in the study are included in the article/supplementary materials, further inquiries can be directed to the corresponding author/s at: xiaoyanmeng@aliyun.com.

## Ethics Statement

Ethical review and approval was not required for the study on human participants in accordance with the local legislation and institutional requirements. The patients/participants provided their written informed consent to participate in this study.

## Author Contributions

X-yM: performance of the research and writing of the paper. JY: performance of the research and data analysis. L-lD: performance of the research and manuscript revise. X-dY and J-aX: performance of the research. J-fW: research design and final approvement. All authors contributed to the article and approved the submitted version.

## Funding

This study was supported by the National Natural Science Foundation of China (82072147) and the Youth Doctor Project of Shanghai Medical Star in the Shanghai Municipal Health Commission.

## Conflict of Interest

The authors declare that the research was conducted in the absence of any commercial or financial relationships that could be construed as a potential conflict of interest.

## Publisher's Note

All claims expressed in this article are solely those of the authors and do not necessarily represent those of their affiliated organizations, or those of the publisher, the editors and the reviewers. Any product that may be evaluated in this article, or claim that may be made by its manufacturer, is not guaranteed or endorsed by the publisher.
